# Bi- or multiparametric MRI in a sequential screening program for prostate cancer with PSA followed by MRI? Results from the Göteborg prostate cancer screening 2 trial

**DOI:** 10.1007/s00330-021-07907-9

**Published:** 2021-04-23

**Authors:** Jonas Wallström, Kjell Geterud, Kimia Kohestani, Stephan E. Maier, Marianne Månsson, Carl-Gustaf Pihl, Andreas Socratous, Rebecka Arnsrud Godtman, Mikael Hellström, Jonas Hugosson

**Affiliations:** 1 Department of Radiology, Institute of Clinical Sciences, Sahlgrenska Academy, University of Gothenburg, Bruna Stråket 11B, 413 45 Gothenburg, Sweden; 2 Department of Radiology, Region Västra Götaland, Sahlgrenska University Hospital, Gothenburg, Sweden; 3 Department of Urology, Institute of Clinical Sciences, Sahlgrenska Academy, University of Gothenburg, Gothenburg, Sweden; 4 Department of Urology, Region Västra Götaland, Sahlgrenska University Hospital, Gothenburg, Sweden; 5 Department of Pathology, Institute of Biomedicine, Sahlgrenska Academy, University of Gothenburg, Gothenburg, Sweden

**Keywords:** Multiparametric magnetic resonance imaging, Prostatic neoplasms, Early detection of cancer, Contrast media

## Abstract

**Objectives:**

The PIRADS Steering Committee has called for “higher quality data before making evidence-based recommendations on MRI without contrast enhancement as an initial diagnostic work up,” however, recognizing biparametric (bp) MRI as a reasonable option in a low-risk setting such as screening. With bpMRI, more men can undergo MRI at a lower cost and they can be spared the invasiveness of intravenous access. The aim of this study was to assess cancer detection in bpMRI vs mpMRI in sequential screening for prostate cancer (PCa).

**Methods:**

Within the ongoing Göteborg PCa screening 2 trial, we assessed cancer detection in 551 consecutive participants undergoing prostate MRI. In the same session, readers first assessed bpMRI and then mpMRI. Four targeted biopsies were performed for lesions scored PIRADS 3–5 with bpMRI and/or mpMRI.

**Results:**

Cancer was detected in 84/551 cases (15.2%; 95% CI: 12.4–18.4) with mpMRI and in 83/551 cases (15.1%; 95% CI: 12.3–18.2%) with bpMRI. The relative risk (RR) for cancer detection with bpMRI compared to mpMRI was 0.99 (95% one-sided CI: > 94.8); bpMRI was non-inferior to mpMRI (10% non-inferiority margin). bpMRI resulted in fewer false positives, 45/128 (35.2%), compared to mpMRI, 52/136 (38.2%), RR = 0.92; 95% CI: 0.84–0.98. Of 8 lesions scored positive only with mpMRI, 7 were false positives. The PPV for MRI and targeted biopsy was 83/128 (64.8%) for bpMRI and 84/136 (61.8%) for mpMRI, RR = 1.05, 95% CI: 1.01–1.10.

**Conclusions:**

In a PSA-screened population, bpMRI was non-inferior to mpMRI for cancer detection and resulted in fewer false positives.

**Key Points:**

*• In screening for prostate cancer with PSA followed by MRI, biparametric MRI allows radiologists to detect an almost similar number of prostate cancers and score fewer false positive lesions compared to multiparametric MRI.*

*• In a screening program, high sensitivity should be weighed against cost and risks for healthy men; a large number of men can be saved the exposure of gadolinium contrast medium by adopting biparametric MRI and at the same time allowing for a higher turnover in the MRI room.*

**Supplementary Information:**

The online version contains supplementary material available at 10.1007/s00330-021-07907-9.

## Introduction

The MRI-based diagnostic pathway for suspected prostate cancer (PCa) has been shown to yield more clinically significant cancers and to reduce overdiagnosis of low-risk cancers compared to systematic biopsies without preceding MRI, and is now being widely implemented as part of clinical routine [1, 2]. Ongoing studies, such as the Göteborg 2 trial, evaluate if the MRI pathway could also balance benefits and harms of population-based PSA screening with special attention to reducing overdiagnosis.

Using MRI as a triage test for men with elevated PSA levels translates to an increased workload for MRI technicians and radiologists. Care needs to be taken to limit both MRI room turnover time and reading time. Consequently, there is an increasing interest in performing biparametric MRI (bpMRI) without dynamic contrast-enhanced imaging (DCE), as opposed to the full multiparametric MRI (mpMRI) protocol recommended by the Prostate Imaging Reporting and Data System (PIRADS) guidelines.

By adopting bpMRI, examination acquisition times can be reduced to less than 15 min, imaging becomes non-invasive, the cost of contrast media is saved, and reading time may be reduced [3–5]. Two recent meta-analyses show that the use of bpMRI does not significantly reduce cancer detection [6, 7]. In response to the increasing interest in performing bpMRI, the PIRADS Steering Committee has issued a consensus statement, calling for “higher quality data before making evidence-based recommendations about bpMRI as an initial diagnostic approach.” However, in a low-risk scenario such as screening, where many men have indolent PCa and overdiagnosis is a major concern, the committee considers bpMRI as a reasonable option [8].

Another recent argument in favor of bpMRI has been concerns about the long-term safety of MRI contrast agents. It has been shown that small amounts of gadolinium can be retained in the brain and other tissues. Although no negative effects have been proven in patients with normal renal function from use of current macrocyclic contrast media in clinical routine, it can be argued from a safety aspect that MRI contrast agents should be used only when they add significant diagnostic value [9].

The aim of this study was to compare the cancer detection rate of bpMRI to that of mpMRI in a low-risk setting (PSA-screened population) by allowing readers to, in the same session, first assess bpMRI and then assess the full mpMRI and note any extra lesions, with targeted biopsies as reference standard.

## Materials and methods

### The Göteborg PCa screening 2 trial design

**Fig. 1:**
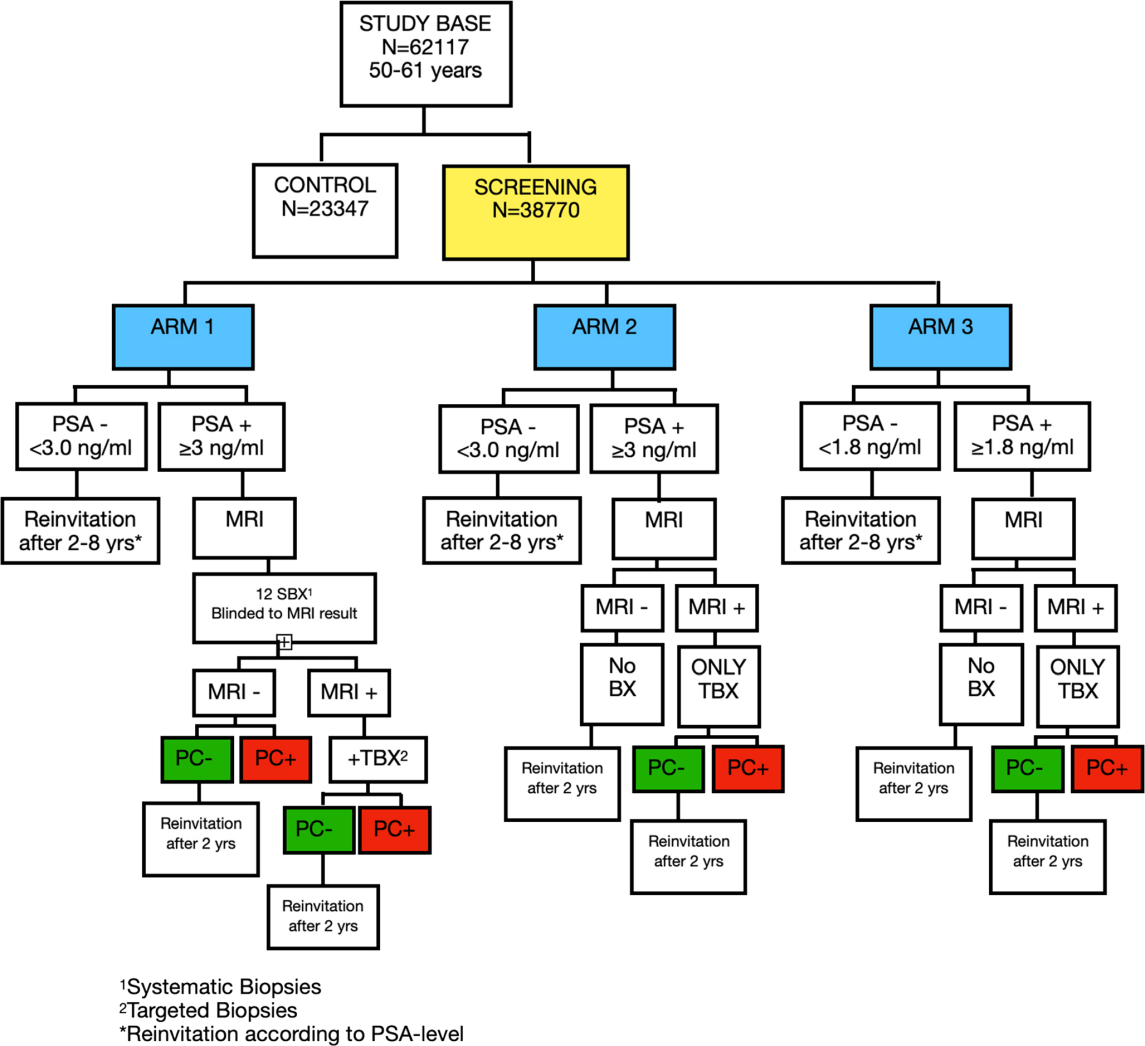
Overview Göteborg Prostate Cancer Screening 2 Trial

The Göteborg PCa screening 2 trial started in 2015 and is an ongoing, long-term single-center study inviting men in the age group 50–61 years in Göteborg and surrounding municipalities to PSA testing followed by mpMRI in case of elevated PSA. From the population registry, approximately 58000 men were randomized to screening invitation or control group. Participating men are further allocated to one out of three study arms according to protocol. Arm 1 is the reference arm in which all men with PSA above the cutoff undergo systematic biopsies (SBX) as well as cognitive-targeted biopsies (TBX) in case of positive MRI. In arms 2 and 3, only men with a positive MRI—defined as PIRADS 3–5—are biopsied and only with cognitive-targeted biopsies (if not PSA > 10 ng/mL, in which case also systematic biopsies are performed). In arms 1 and 2, the PSA cutoff for inclusion is 3 ng/mL, and in arm 3, the PSA cutoff is 1.8 ng/mL. Participants who are not diagnosed with cancer are invited for repeat PSA testing after 2–8 years according to study protocol. An overview of the study design is presented in Fig. 1.

Ethical approval by the Ethical Review Board in Gothenburg was obtained in 2015 (registration number 890-14). The trial is registered as ISRCTN94604465.

### MRI, review, targeted biopsies, and pathological assessment

Imaging was performed using a Philips Achieva dStream 3T MRI with a phased array body coil. Pre-examination preparation included 4 h of fasting and a micro enema 2 h prior to imaging. Multiparametric MRI (mpMRI) was performed compliant to the PIRADSv2 guidelines [10]—including T2W, DWI, and DCE. For DWI (TE, 79 ms), *b*-values of 0, 100, 1000, and 1500 s/mm^2^ were acquired during a 4-min scan with 6-fold averaging along each of three orthogonal encoding directions. Two-fold parallel acceleration was employed to minimize distortions. For calculation of the ADC map, b0 was excluded. Gadolinium contrast medium (Clariscan, 0.5 mmol/mL, GE Healthcare) 0.1 mmol/kg was given via a power injector at a rate of 3 mL/s. DCE images were acquired during 2.5 min with a temporal resolution of 10 s. Total mpMRI acquisition time was 19.1 min.

Three consultant radiologists with 8, 7, and 7 years of experience of prostate MRI, respectively, were readers in this study. Cases were assigned weekly to the available readers on site. Each case was read by two readers in consensus. Information about PSA levels, past medical history, and—if applicable—previous imaging or biopsies was withheld. Reporting was done according to PIRADSv2, with the updated 2.1 version in use after 1 June 2019. Lesions were localized on a 24-sector prostate map template adopted nationally in Sweden, developed in consensus by experts in radiology, urology, and pathology (Supplementary Figure 4).

**Fig. 2:**
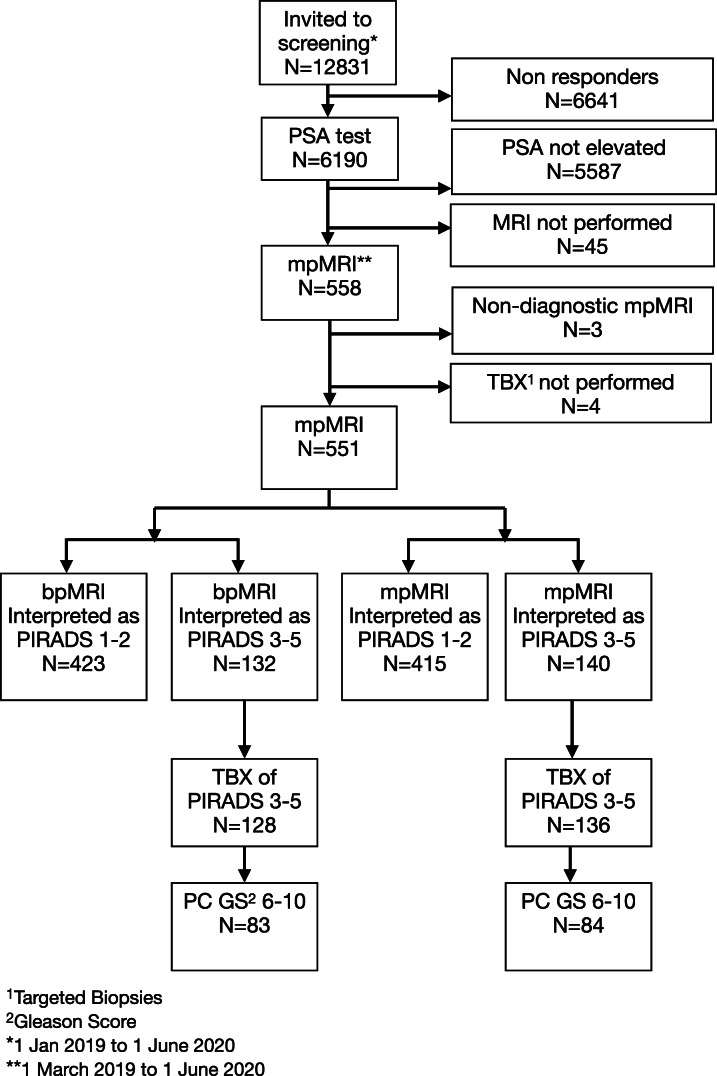
Flow diagram

To assess the performance of bpMRI vs mpMRI for cancer detection, consecutive MRI cases during the time period 1 March 2019 to 1 June 2020 were prospectively read (Fig. 2). The readers first assessed T2-weighted images (in all three imaging planes) and axial DWI/ADC, noting any suspicious MRI lesions on the structured reporting template and designated overall PIRADS scores according to bpMRI for up to three lesions. Subsequently, the contrast medium-enhanced sequence was unblinded and the readers were allowed to assess the full multiparametric MRI, noting any additional lesions and revising PIRADS scores according to contrast enhancement when needed (DCE upgrading). All lesions scored as PIRADS 3 or higher in bpMRI and/or mpMRI were biopsied using a targeted approach.

Targeted transrectal ultrasound (TRUS)-guided biopsies (4 cores/lesion), based on the MRI findings, were directed by cognitive technique (visual registration per sextant). TRUS was performed with a BK medical BK3000 ultrasound machine using a 10 MHz endorectal probe. A limited number (*n* = 5) of experienced urologists (5–30 years of experience) performed the biopsies. Histopathological assessment of biopsy cores was performed by a pathologist (C.-G.P.) specialized in prostate pathology with 25 years of experience in the field.

### Statistical analysis

All analyses were performed on a per patient level. Cancer detection rates (number of men who were diagnosed with PC at cognitive TBX/total number of men who underwent MRI) and false positive rates (number of men with PIRADS 3–5 and negative cognitive TBX/total number of men subjected to cognitive TBX) were calculated and tabulated for bpMRI and mpMRI, accompanied by 95% confidence intervals (CIs). MRI scores were dichotomized and tabulated as positive if overall PIRADS assessment was equal to 3 or greater.

The sample size to achieve 80% power in non-inferiority testing of the limit 0.90 (10% non-inferiority margin) for the ratio of detection rates between bpMRI and mpMRI and a significance level of 5% was calculated. For the comparison of rate of detection of PC of Gleason grade 6–10 a sample size of 550 MRI examinations was needed, given 97% concordant results (MRI positive or negative) between bpMRI and mpMRI and a true positive rate of 15% (number of men with positive cognitive TBX/total number of cognitive TBX).

The statistical analyses were carried out using the Statistical Package for the Social Sciences (SPSS®) version 26 (SPSS Inc., IBM Corp.) and R Statistical Software (version 3.6.1; Foundation for Statistical Computing). The R package ratesci was used for the calculation of CIs (the mid-p method for proportions and the score method for ratios of proportions). The CIs for the ratios of false positive rates and positive predictive values were derived by means of bootstrapping (10000 samples).

## Results

Between 1 March, 2019, and 1 June, 2020, during the first round of screening, mpMRI was performed in 558 men (Fig. 2). Three out of the 558 mpMRI examinations (0.5%) were excluded due to non-diagnostic quality, i.e., artifacts from hip implants or rectal gas. Four MRIs scored PIRADS 3–5 were excluded because TBX were not performed (3 men declined biopsy and 1 man was not biopsied due to comorbidity). The final study cohort is shown in Table 1.

**Table 1:** Patient cohort

*n* = 551	*n*	Median	Interquartile range (IQR)
Age, years	551	57.0	53.8 – 60.0
PSA level, ng/mL	551	3.3	2.3 – 4.5
PSA 1.8–2.9 ng/mL	224		
PSA 3–10 ng/mL	307		
PSA > 10 ng/mL	20		
MRI prostate volume, cm^3^	551	41	33.0 – 53.0
PSA density ng/mL/cm^3^	551	0.075	0.057 – 0.110b

The PIRADS score distributions for bpMRI and mpMRI are shown in Tables 2 and 3. The rate of negative MRI (PIRADS 1–2) was 423 out of 551 (77%) for bpMRI and 415 out of 551 (75%) for mpMRI. The rate of positive MRI (PIRADS 3–5) was 128 out of 551 (23%) for bpMRI and 136 out of 551 (25%) for mpMRI.

**Table 2:** PIRADS distribution bpMRI vs mpMRI. Overall PIRADS score

	1–2	3	4	5	3–5	Total
bpMRI	423 (77%)	59 (11%)	51 (9.3%)	18 (3.3%)	128 (23%)	551
mpMRI	415 (75%)	33 (6.0%)	85 (15%)	18 (3.3%)	136 (25%)	551

**Table 3:** PIRADS concordance bpMRI vs mpMRI. mpMRI overall PIRADS score

		1–2	3	4	5	Total
bpMRI overall PIRADS score	1–2	*415*	**0**	**8**	**0**	423
	3	xx	*33*	**26**	**0**	59
	4	xx	xx	*51*	**0**	51
	5	xx	xx	xx	*18*	18
	Total	415	33	85	18	551

Bold entries indicate mpMRI higher score, italicized entries indicate mpMRI/bpMRI concordant score, xx indicates not applicable in this study design

**Fig. 3:**
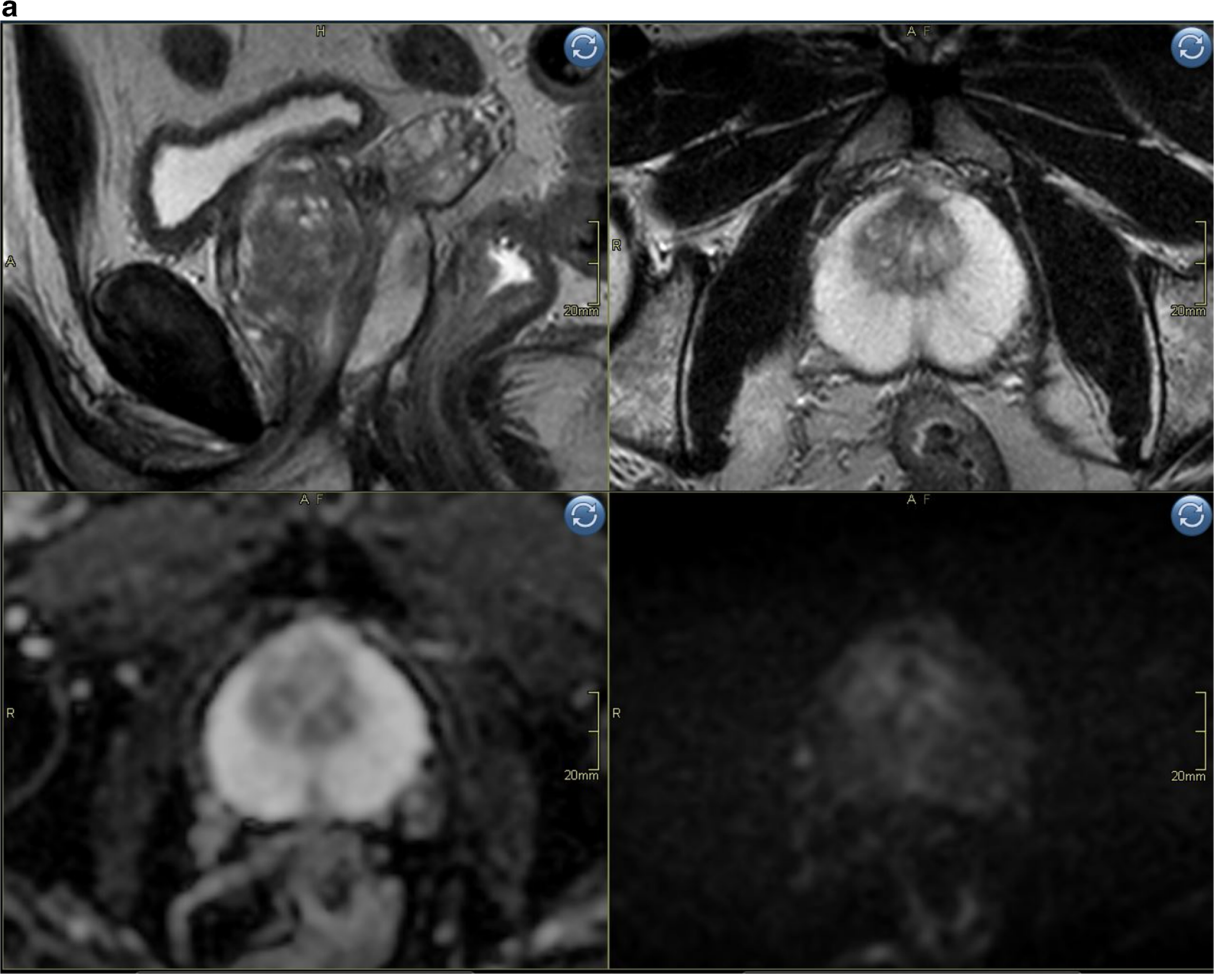

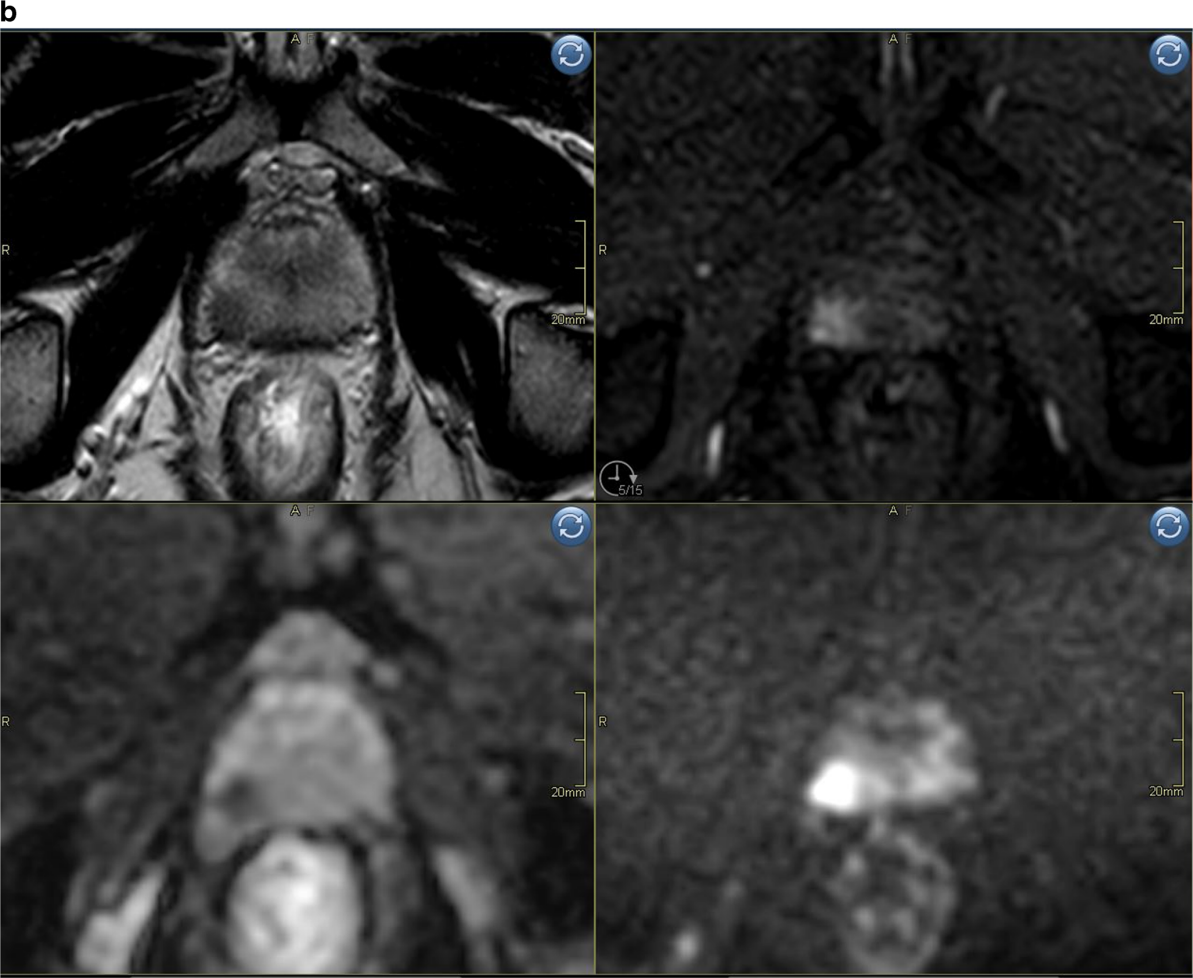

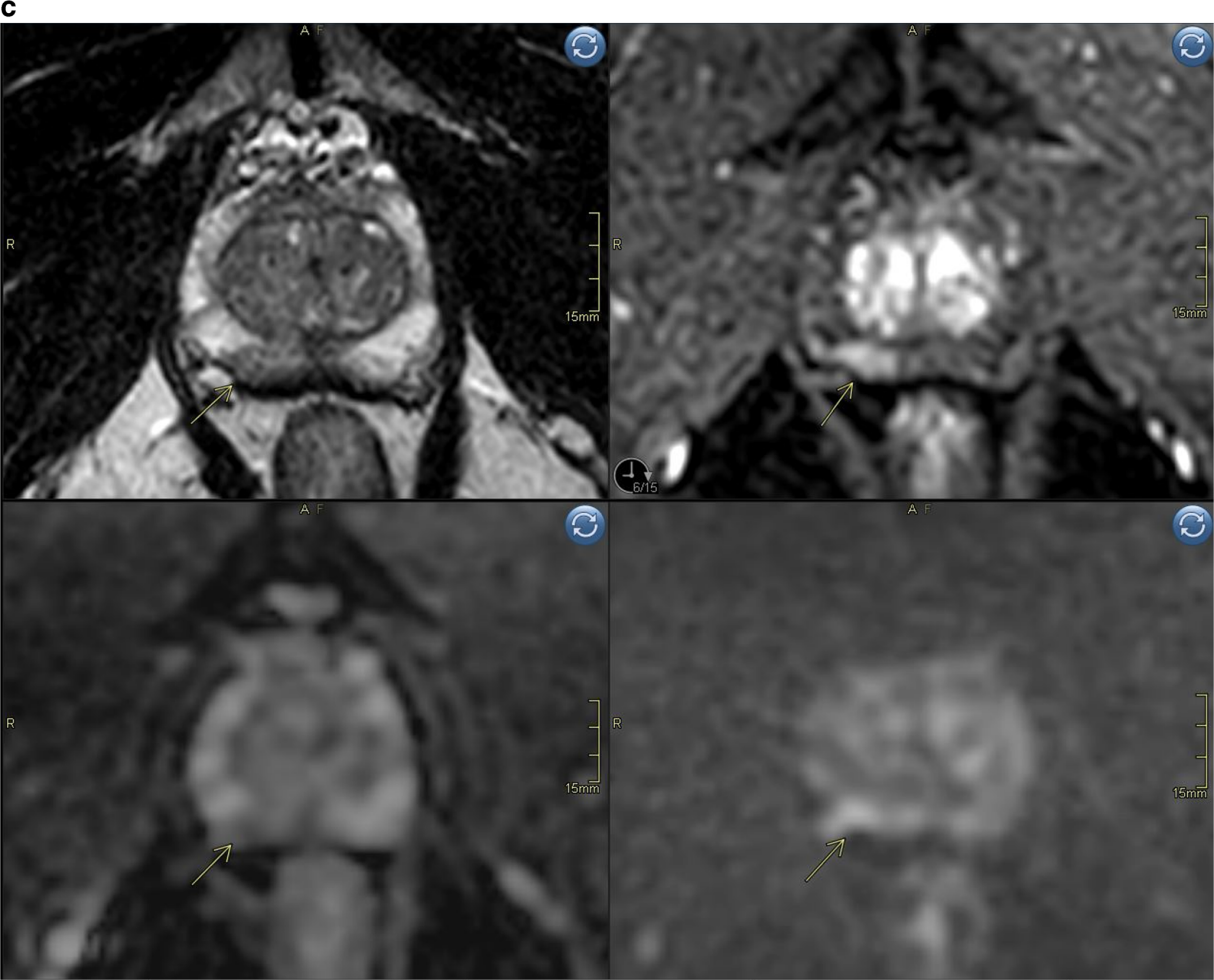
**a** Biparametric MRI example case PIRADS 1 to show image quality. Top left sagittal T2, top right axial T2, bottom left ADC-map, bottom right high b-value (acquired b1500). **b** Example case PIRADS 4 detected both with bpMRI and mpMRI, no additional value of DCE for tumor detection. Top left axial T2, top right axial DCE, bottom left ADC-map, bottom right DWI b1500. Index lesion in the peripheral zone on the right side with markedly restricted diffusion, early contrast enhancement and size < 1.5 cm.  **c** Example case illustrating a false positive case scored PIRADS 2 (DWI = 2) with bpMRI and PIRADS 4 (DWI = 3; DCE = pos) with mpMRI. Top left axial T2, top right axial DCE, bottom left ADC-map, bottom right high b-value DWI (b1500). Periheral zone index lesion on the right side (yellow arrow)

Reading mpMRI resulted in scoring of 8 extra peripheral zone lesions with a PIRADS score of 4 (DWI = PIRADS 3, DCE = positive) (Fig. 3). No additional lesions with a PIRADS score of 3 or 5 were detected only with mpMRI, or any additional transition zone lesions. Seven out of the 8 lesions (88%) detected by mpMRI only were of small size at MRI (long axis < 10 mm) and had low PSAD (< 0.1). Complete characteristics of lesions detected by mpMRI only are shown in Supplementary tables 4a/b.

Out of 84 histology-proven cancers, one cancer was detected only with mpMRI (Table 4). Overall, cancer detection rate was 83 out of 551 (15.1%; 95% CI: 12.3–18.2) with bpMRI and 84 out of 551 (15.2%; 95% CI: 12.4–18.4) with mpMRI. The relative risk (RR) for cancer detection with bpMRI compared to mpMRI was 0.99 (95% one-sided CI: > 94.8 which corresponds to 90% two-sided CI: 94.8–102). With a predetermined 10% non-inferiority margin bpMRI was hence considered non-inferior to mpMRI since the limit of the one-sided CI was 94.8 (Table 5).

**Table 4:** Frequency of biopsy-verified prostate cancers (Gleason score 6–10) stratified by PIRADS score with bpMRI/mpMRI

	bpMRI cancer	mpMRI cancer
PIRADS 1–2	XX/423	XX/415
PIRADS 3	20/59 (34%)	13/33 (39%)
PIRADS 4	46/51 (90%)	54/85 (64%)
PIRADS 5	17/18 (94%)	17/18 (94%)
PIRADS 3–5	83/128 (65%)	84/136 (62%)

**Table 5:** Prostate cancers verified by biopsy (Gleason score 6-10) in lesions scored as PIRADS 3–5 bpMRI vs mpMRI

	bpMRI	mpMRI	RR bpMRI/mpMRI
Gleason score 6–10 PC at biopsy	83/551 = 15.1% (95% CI: 12.3–18.2%)	84/551 = 15.2% (95% CI: 12.4–18.4%)	83/84 = 0.99 (95% CI: 94.8–<->; 90 two-sided CI: 94.8–102)

The false positive rate of bpMRI was 45 out of 128 (35.2%), compared to mpMRI, 52 out of 136 (38.2%), RR = 0.92; 95% CI: 0.84 to 0.98. Out of 8 lesions scored positive by mpMRI only, 7 (88%) were false positives, i.e., showed no cancer at biopsy.

The positive predictive value for the MRI pathway (MRI and cognitive TBX) was 83/128 (64.8%) for bpMRI and 84/136 (61.8%) for mpMRI (Table 4); RR = 1.05, 95% CI 1.01–1.10.

## Discussion

Our study shows that in screening for prostate cancer, PIRADS readers detect an almost equal number of cancers with bi- and multiparametric MRI (83 vs 84 cases, respectively). Several studies based on clinical patients and two recent meta-analyses have reported results very close to ours. In a meta-analysis by Kang et al [7] including a total of 10 studies, 8 retrospective (3146 lesions in total) and 2 prospective (273 lesions in total) similar diagnostic accuracy were reported for bpMRI and mpMRI. In a meta-analysis by Niu et al [6] including 33 studies, the diagnostic accuracy of bpMRI was shown to be high, although the heterogeneity of included studies was substantial. In a retrospective study by Kuhl et al [11], 542 men with mpMRI follow-up of negative systematic biopsies 4 readers with 2–9 years of experience reviewed an abbreviated bpMRI protocol (only trans-axial T2 and DWI images) and then assessed the full mpMRI. The full mpMRI allowed detection of an additional 10 (out of a total of 329) cancers, however, only one significant cancer with a Gleason score of 7. In a prospective study by Zawaideh et al [12] including 264 men, bpMRI identified 116/117 cancers detected by mpMRI with a cutoff of Likert score 3 or greater.

In PSA screening for PCa, approximately one-tenth of men aged 50–60 years will have an elevated PSA (> 3 ng/mL) [13, 14] and the detection rate of cancer is much lower compared to men with a clinical suspicion of PCa where the prevalence of significant cancer many times lies in the range of 30–40% [2]. This means that a larger proportion will have a negative MRI when a screening cohort is examined. The absolute reduction in acquisition time is modest with bpMRI compared to mpMRI, if only considering that contrast wash-in DCE could be performed in 1 min, but the biggest gains are from improved logistics. Using our current mpMRI protocol, the acquisition time is 19 min and the scheduled MRI room turnover rate is 45 min including intravenous access. In the second round of screening, we have adopted bpMRI with an acquisition time of 17 min and room turnover time is now 30 min allowing for at least 5 extra patients per 8-h day. Although we did not record reading times in this study, it is our experience that reading bpMRI takes significantly less time compared to reading mpMRI. This is especially important in a screening program where a high sensitivity should be weighed against costs and risks for healthy men. According to our results, using bpMRI instead of mpMRI would have saved 551 men the exposure of gadolinium contrast medium at the expense of missing one cancer and at the same time allowing for a higher turnover in the MRI room.

We also noted that mpMRI was associated with a higher number of false positive findings; 7 out of 8 lesions scored only by mpMRI to be PIRADS 3–5 were false positive. Our results are in line with the study by Kuhl et al who reported more false positives in mpMRI (43 patients) compared to abbreviated bpMRI (33 patients). Interestingly, in the study by Zawaideh et al using Likert scoring instead of PIRADS, fewer false positives were reported using mpMRI compared to bpMRI (15 vs 27 patients). Likert scoring is different from PIRADS since it allows for DCE downgrading, whereas PIRADS only allows for upgrading. In our study, all of the false positives with mpMRI were indeterminate peripheral zone lesions upgraded based on positive DCE to category 4 (“significant cancer is likely”) according to the PIRADSv2.1 syllable with a recommendation to biopsy. Thus, it can be questioned if the DCE upgrading rules as specified in PIRADSv2 are suitable in a screening scenario. Previous studies validating the concept of DCE upgrading have either been done in prostatectomy cohorts or cohorts of men with clinical suspicion of PCa and thus having a higher overall prevalence of significant cancer (higher pre-test probability) [12, 15–17]. More studies are needed to assess both the value of DCE upgrading and what the optimal follow-up strategy is for lesions with negative biopsy in the low prevalence setting of screening.

Reducing the number of false positive MRI lesions translates to fewer men undergoing biopsies. This is important as these men are spared the discomfort of an invasive procedure—with a known associated 2–3% risk of serious infection requiring hospitalization in the case of transrectal biopsies [18]. Considering that participating men are presumably healthy and asymptomatic, the psychological benefits from not having to worry about what the biopsies might show should also be considered [19]. The biopsy technique used in our study was cognitive targeted which means software-assisted fusion was not used. No clear advantage has been demonstrated using fusion biopsies compared to cognitive biopsies [20].

The PPV of bpMRI compared to mpMRI pathways was slightly in favor of bpMRI (65% vs 62%). The PPV in our study is thus in line with what could be expected in a low prevalence population according to the 2019 Cochrane review [2].

There are limitations to this study. First, imaging was performed at a single site and the readers had high-volume experience including over 1000 consensus readings. Our group of radiologists is likely to have a lower inter-reader variability compared to other groups and the results are not necessarily transferable to radiologists working in low-volume centers using bpMRI. Reader experience has been implicated as a factor in the performance of bpMRI; Gatti et al [21] reported that less-experienced readers (< 1000 cases) benefitted from mpMRI in index lesion detection. However, Di Campli et al did not find that reader experience significantly modified the diagnostic performance of both MRI protocols [22]. High volume alone is however not sufficient. Quality control including feedback to MRI technicians, inter-reader feedback, feedback from biopsies, and an ongoing discussion between radiology, urology, pathology, and oncology is of paramount importance in the making of a successful prostate MRI pathway [23]. A future screening program would likely be a highly specialized, multi-disciplinary effort.

Second, as only consensus readings were performed, we did not measure inter-reader variability. Many studies have reported moderate inter-observer agreement in PIRADS assessment [24, 25]. We therefore choose consensus reporting in order to try to reduce variability. The reporting template was filled in by two experienced readers in consensus and thus no data on inter-reader variability is available. We acknowledge that consensus reading does not reflect clinical practice in most institutions, but reasonably it does not hamper the comparison between bpMRI and mpMRI in the present study, as consensus reading was made for both bpMRI and mpMRI

Third, unblinding DCE in the same reading session can cause a bias to under- or over-report findings on DCE depending on personal preferences. However, we followed the pattern we believe most readers use, viewing DCE last to confirm findings on T2 and DWI and to look for additional findings. Performing a full randomized trial of reading bpMRI vs mpMRI in screening would require a very large sample size of approximately 12600 MRIs even with a non-inferiority limit of 10% (assuming equal detection rate bpMRI/mpMRI, 15% PCa prevalence, 80% power, and 5% significance level). Performing such a study seems hard to motivate given the small reported effect size of DCE on cancer detection.

Fourth, it should be emphasized that biparametric MRI (Fig. 3) relies heavily on the quality of the diffusion-weighted sequence (DWI) which can be prone to artifacts, requiring good patient preparation and attentive MRI technicians. Our 3T protocol was optimized for high lesion conspicuity within a clinically practical scan time. This involved the acquisition of a b1500 image with three encoding directions and six-fold averaging. A three-point ADC analysis based on b100, b1000, and b1500 was used to maximize the SNR of the ADC map. This may result in slightly different ADC values than a fit with a low and intermediate *b*-value alone, as suggested by the PIRADS guidelines. However, this limitation should not be overemphasized, since the ADC values were not used quantitatively and since tissue ADC is also determined by other parameters, such as echo time and diffusion time, which in turn are determined by protocol settings and system performance. Thus, setting just equal *b*-values does not necessarily result in reproducible ADC values. Only if we use exactly the same system specifications with exactly the same protocol parameters can we expect reproducible tissue ADC values. Obtaining the high *b*-value with a separate acquisition is preferred but not mandatory, according to PIRADS guidelines/recommendations [10]. Some groups consider using (monoexponentially) extrapolated ultra-high *b*-values ≥ 2000 mm/s^2^ to be beneficial in order to increase reader confidence [26]. According to PIRADS guidelines/recommendations, the use of computed high *b*-values is optional. We did not include computed ultra-high *b*-values in the present study. Finally, a 1.5T platform may require different protocol settings and may have resulted in slightly different findings. A well-performed 1.5T examination may in fact, in certain aspects, provide better images, e.g., in terms of geometric distortions. Differences in protocols across institutions may influence the results of bpMRI in general, but the effect size is likely small as long as protocols include a PIRADS-compliant high *b*-value and are reasonably well optimized.

Fifth, in this report, we do not present data on Gleason grading since these data are censored by recommendation of the study data monitoring committee before analysis of the main study endpoints. The reported detection rate refers to cancer of any Gleason grade (Gleason 6–10), some of which might be low grade and of limited clinical importance.

## Conclusion

In screening for PCa with PSA followed by 3T MRI, experienced PIRADS readers detected an almost equal number of prostate cancers and scored fewer false positive lesions using bpMRI, as compared to mpMRI. However, future bpMRI protocols will certainly benefit from DWI protocols with fully reproducible quantitative ADC values. In conclusion, bpMRI seems advantageous to mpMRI due to similar detection rate, lower rate of false positives, shorter examination time, lower cost, and no potential harms of gadolinium contrast medium administration.

## Supplementary Information

ESM 1 (DOCX 113 kb)

## Abbreviations

bpMRI: Biparametric MRI
DCE: Dynamic contrast enhancement
DWI: Diffusion-weighted imaging
GS: Gleason score
mpMRI: Multiparametric MRI
MRI: Magnetic resonance imaging
PCa: Prostate cancer
PIRADS: Prostate Imaging Reporting and Data System
PSA: Prostate-specific antigen
PSAD: Prostate-specific antigen density
SBX: Systematic biopsies
TBX: Targeted biopsies
TRUS: Transrectal ultrasound

## Declarations

### Guarantor

The scientific guarantor of this publication is Professor Jonas Hugosson.

### Statistics and biometry

One of the authors, Marianne Månsson, associate professor of statistics has significant statistical expertise.

### Methodology

• Prospective

• Diagnostic or prognostic study

• Performed at one institution
