# Supplementary material for: Bi- or multiparametric MRI in a sequential screening program for prostate cancer with PSA followed by MRI? Results from the Göteborg prostate cancer screening 2 trial
Source: Eur Radiol. 2021 Apr 23;31(11):8692–702. doi: 10.1007/s00330-021-07907-9 (PMC8523442; doi:10.1007/s00330-021-07907-9)

**Supplementary Materials**

**Table 4a**: Characteristics of false positive lesions only detected by mpMRI

| Size (mm) | PZ^1^/TZ^2^ | DCE | PIRADS | PSAD^3^ |
| --- | --- | --- | --- | --- |
| 7 x 5 | PZ | + | 4 | 0.08 |
| 17 x 5 | PZ | + | 4 | 0.05 |
| 6 x 4 | PZ | + | 4 | 0.26 |
| 7 x 6 | PZ | + | 4 | 0.09 |
| 8 x 7 | PZ | + | 4 | 0.07 |
| 5 x 3 | PZ | + | 4 | 0.11 |
| 8 x 5 | PZ | + | 4 | 0.04 |

^1^Peripheral zone

^2^Transition zone

^3^Prostate Specific Antigen Density

**Table 4b**: Characteristics of true positive lesion only detected by mpMRI

| Size (mm) | Zone | DCE | PIRADS | PSAD |
| --- | --- | --- | --- | --- |
| 6 x 5 | PZ | + | 4 | 0.16 |

**Figure 4**


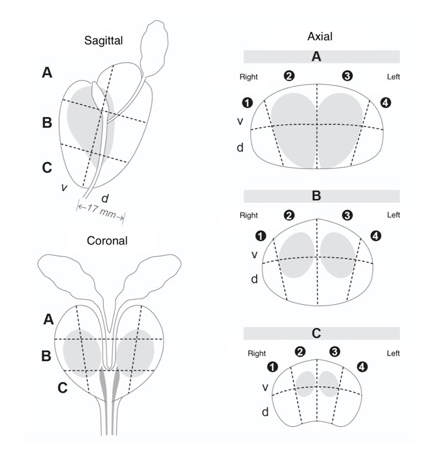

Supplement: Supplementary file 1 — (DOCX 113 kb) [file 330_2021_7907_MOESM1_ESM.docx]
